# Sperm Cells of a Primitive Strepsipteran

**DOI:** 10.3390/insects4030463

**Published:** 2013-09-04

**Authors:** James B. Nardi, Juan A. Delgado, Francisco Collantes, Lou Ann Miller, Charles M. Bee, Jeyaraney Kathirithamby

**Affiliations:** 1 Department of Entomology, University of Illinois, 320 Morrill Hall, 505 S. Goodwin Avenue, Urbana, IL 61801, USA; 2 Department of Zoology and Physical Anthropology, Faculty of Biology, University of Murcia, Murcia 30100, Spain; E-Mails: jdelgado@um.es (J.A.D.); fcollant@um.es (F.C.); 3 Biological Electron Microscopy, Frederick Seitz Materials Research Laboratory, Room 125, University of Illinois, 104 South Goodwin Avenue, Urbana, IL 61801, USA; E-Mail: lamiller@illinois.edu; 4 Imaging Technology Group, Beckman Institute for Advanced Science and Technology, University of Illinois, 405 N. Mathews Avenue, Urbana, IL 61801, USA; E-Mail: c-bee1@illinois.edu; 5 Department of Zoology, South Parks Road, Oxford OX1 3PS, UK; E-Mail: jeyaraney.kathirithamby@zoo.ox.ac.uk

**Keywords:** testis, sperm structure, Strepsiptera, Meloidae, Rhipiphoridae

## Abstract

The unusual life style of Strepsiptera has presented a long-standing puzzle in establishing its affinity to other insects. Although Strepsiptera share few structural similarities with other insect orders, all members of this order share a parasitic life style with members of two distinctive families in the Coleoptera—the order now considered the most closely related to Strepsiptera based on recent genomic evidence. Among the structural features of several strepsipteran families and other insect families that have been surveyed are the organization of testes and ultrastructure of sperm cells. For comparison with existing information on insect sperm structure, this manuscript presents a description of testes and sperm of a representative of the most primitive extant strepsipteran family Mengenillidae, *Eoxenos laboulbenei*. We compare sperm structure of *E. laboulbenei* from this family with that of the three other families of Strepsiptera in the other strepsipteran suborder Stylopidia that have been studied as well as with members of the beetle families Meloidae and Rhipiphoridae that share similar life histories with Strepsiptera. Meloids, Rhipiphorids and Strepsipterans all begin larval life as active and viviparous first instar larvae. This study examines global features of these insects’ sperm cells along with specific ultrastructural features of their organelles.

## 1. Introduction

Sperm of insects show a great diversity of external shapes and sizes; and their internal ultrastructure is correspondingly complex and variable. A basic ground plan exists for the sperm structure of all insects, and specific variations on this basic plan have proved to be useful characters in the study of insect phylogeny. These architectural features of insect sperm offer subcellular characters that may help resolve certain phylogenetic relationships that have stubbornly defied analysis using somatic characters [1,2]. Here we present information on the architecture of sperm cells from not only a primitive family of Strepsiptera but also two families in the Coleoptera. Members of the Coleoptera are now considered to be the closest living relatives of the Strepsiptera.

This manuscript presents a description of sperm cells from a primitive Strepsipteran of the suborder Mengenillidia and family Mengenillidae, *Eoxenos laboulbenei* De Peyerimhof. Only female members of the family Mengenillidae are free-living while in all other strepsipteran families, the females have lost several morphological features and remain endoparasitic except for their cephalothoraces that extrude from their hosts’ integuments. *Eoxenos*, *Mengenilla*, and *Congoxenos* (Mengenillidae) are the only members of the suborder Mengenillidia and are considered the most primitive of extant Strepsiptera [3,4,5].

We compare the sperm structure of *E. laboulbenei* from this primitive family with that of the three other families of Strepsiptera in the suborder Stylopidia studied so far (Xenidae, Elenchidae and Halictophagidae) as well as with members of two families in the Coleoptera—the most closely related neuropteroid order based on recent genomic evidence [6,7]. Based on similarities in the life histories of Strepsiptera and certain beetles, two beetle families in the suborder Polyphaga—Meloidae and Rhipiphoridae—were chosen for comparison with Strepsipteran families. These two beetle families are unique among beetles in sharing a parasitic life style with Strepsiptera. Meloids, Rhipiphorids and Strepsipterans all begin larval life as active, viviparous, planidial first instar larvae [4,8,9,10]. This study compares global features of these insects’ sperm cells along with specific ultrastructural features of their organelles: (1) the flagella/nucleus interface; (2) acrosome/nucleus interface and (3) flagellar architecture revealed in transverse sections.

## 2. Experimental Section

### 2.1. Collection of Insects

Insects were collected in Spain at Landfarm and Lemmon Orchards on the road from Mula to Pliego, Murcia (2°28'52.46'' W 38°00'25.27'' N). Male *E. laboulbenei* (Strepsiptera) were attracted to a light trap consisting of three cross-shaped sheets of plastic with an ultraviolet light attached at the center of the intersections. This device was placed on a white plastic container on a table. Flying males of *E. laboulbenei* were collected directly using an aspirator and were immediately transported to the laboratory in a cooler for fixation.

### 2.2. Preparation of Tissues for Light and Electron Microscopy

Beetles and strepsipterans were dissected in Petri dishes with black Sylgard bottoms. Anesthetized animals were submerged in Grace’s insect culture medium. Tissues used for electron microscopy and for staining of one micron sections with toluidine blue and basic fuchsin were prepared as described in Section 2.2.1 below. Tissues used for squashes and labeling with the fluorescent nuclear stain DAPI (4',6-diamidino-2-phenylindole) were fixed and processed according to the procedure presented in Section 2.2.2.

#### 2.2.1. Preparation of Tissues for Sectioning

A standard fixation procedure for insect tissues was used to prepare testes for sectioning. Specimens for transmission electron microscopy and light microscopy were fixed at 4 °C in a primary fixative of 2.5% glutaraldehyde and 0.5% paraformaldehyde dissolved in a rinse buffer of 0.1 M cacodylate (pH 7.4) containing 0.18 mM CaCl_2_ and 0.58 mM sucrose. After three hours in this fixative, tissues were washed three times with rinse buffer before being transferred to the secondary fixative (2% osmium tetroxide in rinse buffer). Tissues remained in this solution for 4 h in the cold and were then washed three more times with rinse buffer. To enhance membrane contrast, rinsed tissues were placed in filtered, saturated uranyl acetate for 15 min immediately before being gradually dehydrated in a graded ethanol series (10%–100%).

A special fixation procedure that uses tannic acid in the primary fixative and replaces osmium tetroxide with uranyl acetate in the secondary fixative has been used extensively by others to resolve the protofilament organization of individual microtubules of sperm axonemes [1,2,11]. However, the procedure described in the preceding paragraph was used as a method that seems to preserve a wider range of ultrastructural features of sperm cells even though individual protofilaments of axonemes cannot be resolved in the images of this manuscript.

From absolute ethanol, tissues for sectioning were transferred to propylene oxide and infiltrated with mixtures of propylene oxide and resin before being embedded in pure LX112 resin. Resin was polymerized at 40 °C for one day and at 60 °C for three days.

Embedded tissues were sectioned with a diamond knife either at 1.0 µm for light microscopy or at ~0.09 µm for electron microscopy. Sections for light microscopy were floated onto glass slides covered with distilled water. After the water had evaporated, the adherent sections were stained with a solution of 0.5% toluidine blue and 0.25% basic fuchsin in 1% borax. Thin sections of those regions of testes chosen for ultrastructural examination were mounted on copper grids and stained briefly with saturated aqueous uranyl acetate and Luft’s lead citrate to enhance contrast. Images were taken with a Hitachi H600 transmission electron microscope operating at 75 kV.

#### 2.2.2. Preparation of Sperm Whole Mounts

Whole testes used for fluorescent labeling were always fixed in 100% methanol and stored at −20 °C until prepared as squashes. From 100% methanol, tissues were transferred to 50% acetic acid for at least 30 min. After exposure to acetic acid, testes were placed on a glass slide and a cover glass was positioned over the testis. By applying gentle pressure to the cover glass, cells of the testis were dispersed from a three-dimensional configuration into a two-dimensional array. Excess liquid was removed from the edge of the cover glass, and the slide was placed upside down on dry ice for at least two minutes. The cover glass was quickly flipped off the frozen and squashed testis with the edge of a razor blade. Tissue was further permeabilized for at least 30 min by addition of a permeabilization buffer (PBS + 10% normal goat serum + 0.1%Triton X-100). The cells were subsequently stained by addition of a 1:1,000 dilution of 4',6-diamidino-2-phenylindole (DAPI, 1 mg/mL distilled water) to this permeabilization buffer. All nuclei label in cells that are exposed to DAPI. Squashes of testes were mounted in 70% glycerin (*v/v*) in 0.1 M Tris (pH 9.0). Specimens were imaged with a Nikon E600 using a combination of fluorescent and Nomarski optics. Sperm dimensions were measured using ImagePro software (Media Cybernetics).

## 3. Results and Discussion

### 3.1. Testis Organization

Each insect testis consists of one or more sperm tubes or follicles in which the enclosed germ cells are usually aligned. Each mature sperm tube usually contains male germ cells at all stages of spermatogenesis, from spermatogonial stem cells to differentiated sperm.

**Figure 1:**
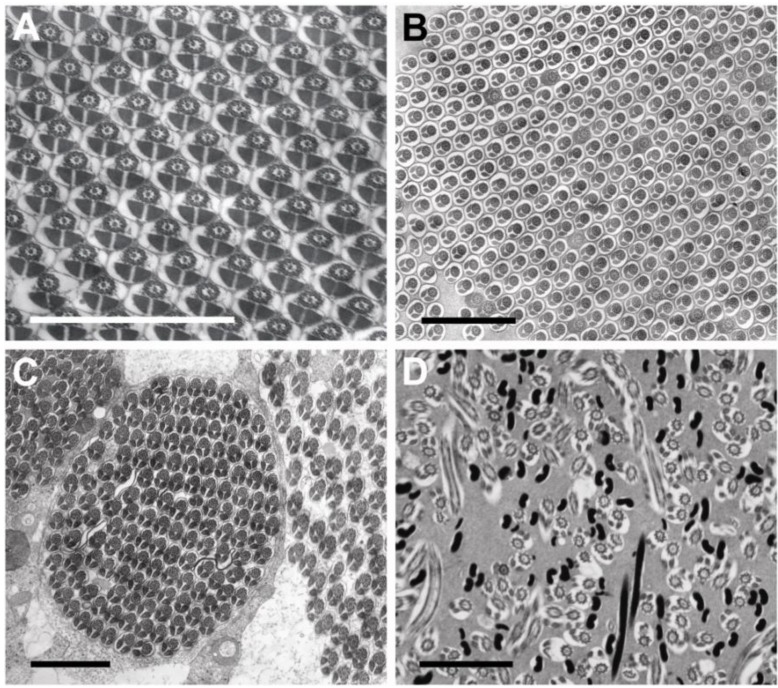
(**A**–**C**) Images show arrays of aligned sperm in coleopteran testes. (**A**) *Mylabris variabilis*; (**B**) *Zonitis flava*; (**C**) *Macrosiagon triscuspidata*; (**D**) *Eoxenos laboulbenei.* This latter image shows the haphazard alignment of strepsipteran sperm. Sections of sperm flagella are intermixed with sections of sperm nuclei. Sections of nuclei are black. Each scale bar = 2.0 μm.

The sperm follicles observed in the two beetle families contain highly ordered, synchronously developing arrangements of sperm cells at various stages of spermatogenesis that resemble crystalline lattices (Figure 1A–C). The rhipiphorid beetle *M. triscuspidata* has 12 loosely adherent sperm tubes per testis. The meloid beetles, *H. scutellatus*, *M. variabilis* and *Z. flava* representing three different genera and the two subfamilies of Meloidae, each have 20 or more tightly adherent sperm tubes per testis. In contrast, all Strepsiptera species examined previously and *E. laboulbenei* examined here have one sperm tube per testis [12]; within this one tube or follicle, all germ cells were represented by mature spermatozoa and did not show the parallel alignment of cells. Instead, in a given section of sperm follicle, cell sections appear at all angles between transverse and longitudinal (Figure 1D).

### 3.2. Sperm Architecture

**Figure 2:**
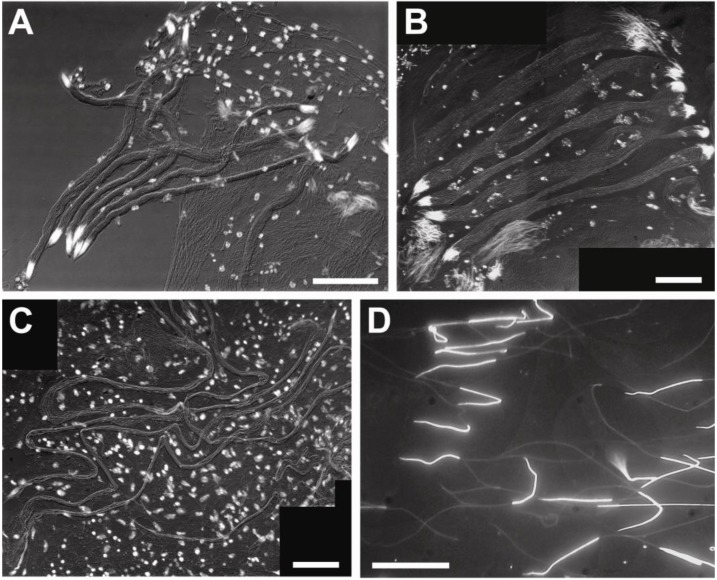
Representative whole mounts of sperm are shown. Sperm were measured in these squashes prepared from testes. Nuclei have been labeled with the fluorescent probe DAPI. Note the organized grouping of beetle sperm in spermatodesmata, and the absence of this organization for the strepsipteran sperm. (**A**) *Hycleus scutellatus*. (**B**) *Mylabris variabilis*. (**C**) *Macrosiagon triscuspidata*. (**D**) *Eoxenos laboulbenei.* Scale bars = 100 μm in **A**, **C**; scale bar = 50 μm in **B**; scale bar = 20 μm in **D**.

The dimensions of sperm heads and tails were measured in squashes prepared from testes of the different species and imaged with fluorescent and differential interference microscopy (Table 1, Figure 2). Sperm lengths ranged from about 55 μm in *E. laboulbenei* (Strepsiptera) to about 1,236 μm in Rhipiphoridae. The lengths of meloid sperm lie between these extremes. The lengths of sperm heads were not always proportional to the total lengths of the sperm; head lengths ranged from about 13 μm for the meloid *Z. flava* to about 33 μm for the rhipiphorid *M. triscuspidata*. These measurements were based on DAPI staining of nuclear DNA.

**Table 1:** Measurements of beetle and strepsipteran sperm cells.

Order, Family	Species, Subfamily	Total Length ± s.d. (μm)	Head Length ± s.d. (μm)	~Ratio of Tail Length:Head Length
Coleoptera, Meloidae	*Zonitis flava* Nemognathina*e*	365.1 ± 22.3 (*n* = 4)	13.3 ± 1.1 (*n* = 9)	~27:1
Coleoptera, Meloidae	*Hycleus scutellatus* Meloinae	362.5 ± 13.4 (*n* = 8)	18.1 ± 3.3 (*n* = 11)	~19:1
Coleoptera, Meloidae	*Mylabris variabilis* Meloinae	603.3 ± 77.1 (*n* = 8)	29.8 ± 4.4 (*n* = 32)	~19:1
Coleoptera, Rhipiphoridae	*Macrosiagon* *tricuspidata* Rhipiphorinae	1,236.3 ± 38.1 (*n* = 4)	32.8 ± 3.3 (*n* = 13)	~37:1
Strepsiptera, Mengenillidae	*Eoxenos* *laboulbenei*	55.3 ± 7.8 (*n* = 6)	17.6 ± 2.2 (*n* = 6)	~2:1

Within testes, beetle sperm are organized in highly ordered arrays of cells aligned in register (spermatodesmata) whereas strepsipteran sperm show an absence of such organization (Figure 1). Not only do beetle sperm and strepsipteran sperm differ in their organization with testes, but also the flagella of meloid and rhipiphorid sperm exceed the flagellar length of *Eoxenos* sperm by factors of between 9 and 15 for Meloidae and about 32 for Rhipiphoridae. Ratios of length of sperm flagellum to length of sperm head show a similar hierarchy. *Eoxenos* sperm have relatively short flagella measuring only about twice the length of the sperm head whereas the length of the sperm tail for *M. tricuspidata* (Rhipiphoridae) measures about 37 times the length of its head. The two members of the subfamily Meloinae have about the same ratios for their sperm heads and flagella whereas *Z. flava* representing the other subfamily of the Meloidae has a greater proportion (27:1) of sperm length devoted to flagellum (Table 1, Figure 2).

Approximate ratios of sperm flagellar length to sperm head length for all strepsipterans examined are all smaller than 10 to 1: 5:1 for *Halictophagus chilensis* [13], 3:1 for *Xenos vesparum* [1], and 2:1 for *Eoxenos laboulbenei*. Each of these species represents a different family of Strepsiptera*. H. chilensis* and *X. vesparum* represent the suborder Stylopidia, and *E. laboulbenei* represents one of the two extant families in its suborder Mengenillidia.

Compared with sperm of rhipiphorid and meloid beetles, *Eoxenos* sperm and sperm cells of these members of the other strepsipteran suborder Stylopidia have relatively short flagella measuring only about twice to five times the length of the sperm head whereas the length of the sperm tails for these beetles measures from about 19–37 times the length of the sperm heads (Table 1).

### 3.3. Sperm Ultrastructure

#### 3.3.1. Sperm Heads: Nuclei and Acrosomes

**Figure 3:**
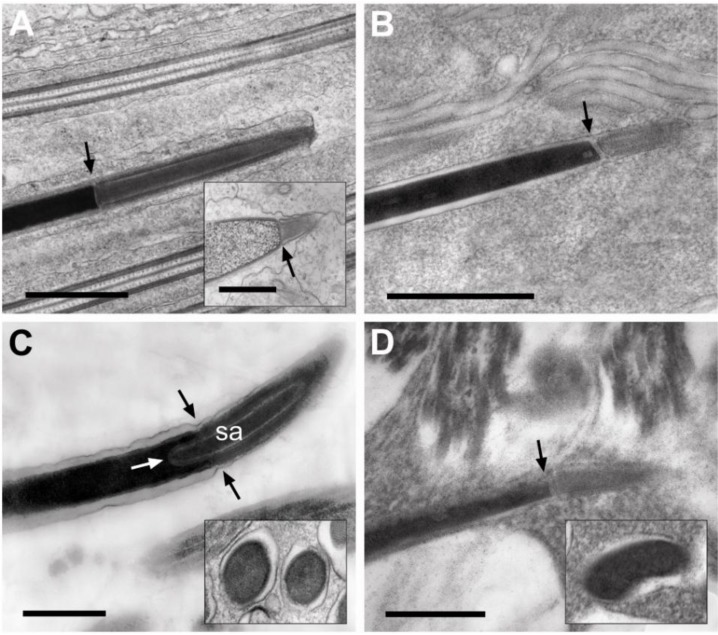
The acrosome forms a straight interface (arrows) with the adjacent nucleus in Meloids and Strepsipteran (**A**, **B**, **D**). The rhipiphorid acrosome, by contrast, with its subacrosomal material (sa) forms a curved interface with the nucleus. (**C**) Its interface with the nucleus is marked with three arrows. The acrosome is at right in all figures. (**A**) *Hycleus scutellatus*. Inset = *Mylabris variabilis.* (**B**) *Zonitis flava.* (**C**) *Macrosiagon triscuspidata*. (**D**) *Eoxenos laboulbenei.* The insets in **C** and **D** show shapes of nuclei in transverse sections for *M. triscuspidata* and *E. laboulbenei,* respectively*.* Scale bars = 1.0 μm in **A**; scale bar = 0.5 μm in **B**; scale bars = 0.25 μm in **C**, **D**.

Multiple transverse and longitudinal sections of spermatozoa and spermatids were examined in this study. The acrosome-nucleus interfaces for the four beetle species and for the strepsipteran are marked with arrows in Figure 3. The acrosomes of the species of Meloids that are illustrated here, *Z. flava* (subfamily Nemognathinae) and *H. scutellatus* (subfamily Meloinae), have a simple organization (Figure 3A,B) and lack the conspicuous subacrosomal material observed in some other beetles (Figure 3C). A longitudinal section of a spermatid acrosome of *M. variabilis* (subfamily Meloinae) also illustrates this simple organization of the acrosome in the Meloinae (inset, Figure 3A). The organization of the rhipiphorid acrosome with its conspicuous subacrosomal material (Figure 3C), however, is clearly an exception to the simple layered arrangement observed for meloid and strepsipteran acrosomes (Figure 3A,B,D); by contrast, each meloid and strepsipteran acrosome has a uniformly dense interior circumscribed by a lighter electron-dense periphery and an outermost electron-dense membrane.

The nuclei of all meloid and rhipiphorid sperm are circular in transverse section (inset, Figure 3C); the elongate nuclei of *E. laboulbenei*, like the nuclei of other strepsipteran sperm [1,13,14,15], are kidney-shaped in cross section (Figure 1D; inset, Figure 3D).

In the ground plan of sperm architecture, the acrosomes of Coleoptera and Strepsiptera have been described as multi-layered and mono-layered respectively [15]. The acrosome of the rhipiphorid *Pelecotoma fennica* (subfamily Pelecotominae) [14]) was described as being three-layered while the acrosome of the meloid *Lytta vesicatoria* (subfamily Meloinae) was described as being two-layered [15]. With the one exception of *Xenos vesparum* examined by Dallai *et al*. [1], all other Strepsiptera examined including other *Xenos* species have been reported to have acrosomes [13,14,16].

#### 3.3.2. Sperm Necks

**Figure 4:**
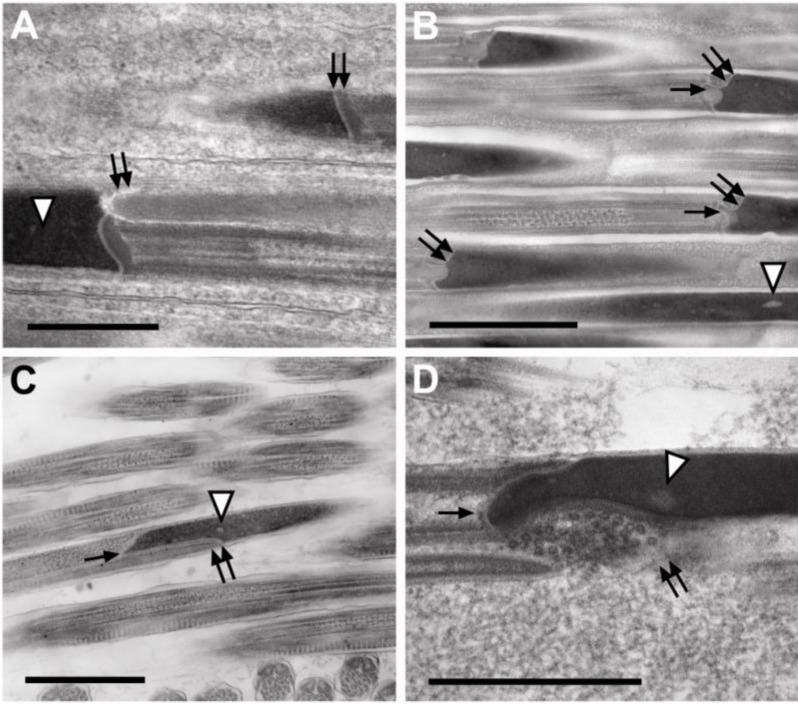
Junction of posterior end of nucleus with axoneme and mitochondrial derivatives. Each single arrow points to posterior process of nucleus that interfaces with the axoneme. Double arrows indicate the interface of sperm nucleus and mitochondrial derivative. Arrowheads point to uncondensed chromatin. (**A**) *Hycleus scutellatus*. (**B**) *Zonitis flava.* (**C**) *Macrosiagon triscuspidata*. (**D**) *Eoxenos laboulbenei.* Scale bars = 1.0 μm in **A**–**C**; scale bar = 0.5 μm in **D**.

The structure of this region shows marked differences between subfamilies in the family Meloidae: *H. scutellatus* represents the subfamily Meloinae and *Z. flava* represents the subfamily Nemognathinae. In the neck region of the sperm, the posterior end of the nucleus forms a straight interface with the flagellum as in *H. scutellatus* (arrows, Figure 4A); or in the case of *Z. flava*, *M. tricuspidata*, and *E. laboulbenei*, the posterior pole of the nucleus is indented at its interface with the anterior end of the mitochondrial derivatives (double arrows, Figure 4B–D). In these three latter images, the interface of the axoneme and the nucleus lies at a more posterior location: about 0.25 μm from the anterior end of the mitochondrial derivatives.

In the case of the sperm of *Z. flava*, *M. tricuspidata*, and *E. laboulbenei*, a process extends posteriorly from the posterior-most end of each nucleus and interfaces with the edge of the axoneme adjacent to the mitochondrial derivatives. These nuclear processes are marked with single arrows in Figure 4B–D. In *E. laboulbenei*, this interface of the nucleus with the mitochondrial derivative of the flagellum (double arrows, Figure 4B) is consistently occupied by a cluster of distinctive vesicles, each measuring approximately 18 nm.

In both the strepsipteran and the two beetle families, the head region of the sperm is a very elongate nucleus containing regions of uncondensed chromatin, the latter indicated with arrowheads in Figure 4A–D.

#### 3.3.3. Sperm Flagella: Axonemes, Mitochondrial Derivatives and Accessory Bodies

**Figure 5:**
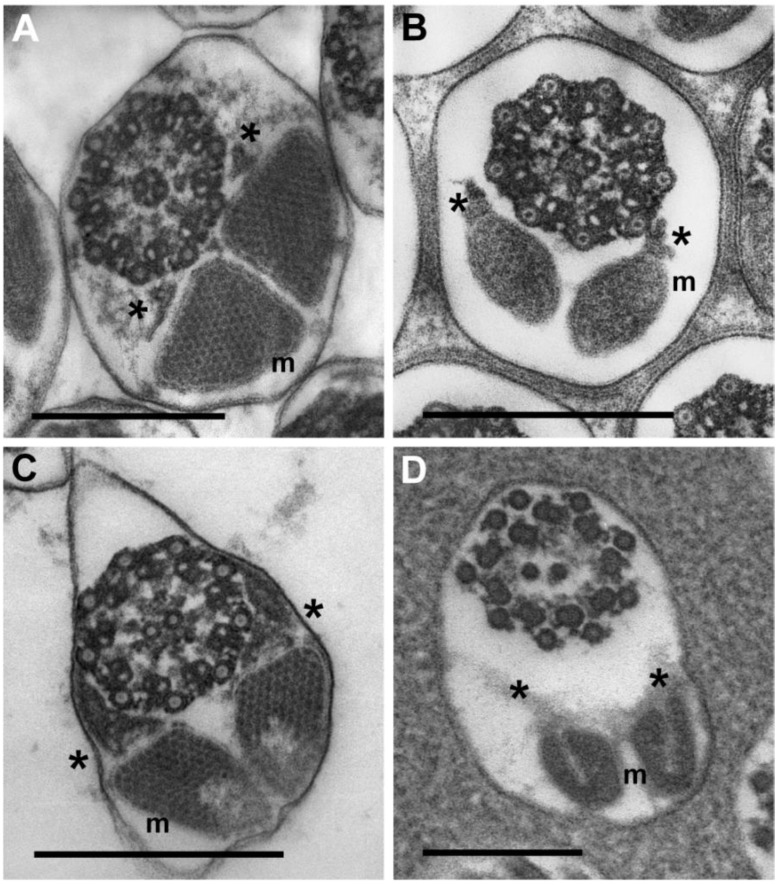
High magnification images of beetle and strepsipteran axonemes, mitochondrial derivatives and accessory bodies. These transverse sections of flagella are aligned so that mitochondrial derivatives (m) lie at the bottom in each image; axonemes lie at the top. Positions of distinct accessory bodies in **B**, **C** or indistinct accessory bodies in **A**, **D** are marked with small asterisks. (**A**) *H.scutellatus* (Meloidae, Meloinae)*.* (**B**) *Z. flava* (Meloidae, Nemognathinae). (**C**) *M. triscuspidata* (Rhipiphoridae, Rhipiphorinae). (**D**) *Eoxenos laboulbenei.* Each scale bar = 0.25 μm.

Many distinguishing characters of insect sperm lie within the flagellum: (1) the axoneme or the microtubule-based cytoskeleton of the flagellum; (2) one or two mitochondrial derivatives; and (3) one, or more commonly two, accessory bodies that represent extensions of the centriole adjunct that forms a collar at the base of the flagellum. Figure 5A–C show two meloid axonemes (A,B) and a rhipiphorid axoneme (C). Features that are not pronounced for the axoneme of *E. laboulbenei* (Figure 5D) but are conspicuous in all the beetle axonemes (Figure 5A–C) are the nine radial links between the two central tubules and the doublet tubules. Intertubular material [1,11] is observed in axonemes of the two beetle families examined as well as in axonemes of *E. laboulbenei.*

Mitochondrial derivatives of the four beetles examined here all contain crystalline inclusions. Each arrangement of inclusions within the mitochondrial derivatives, however, is distinctive (Figure 5A–C, m). Mitochondrial derivatives of *E. laboulbenei*, by contrast, lack conspicuous crystalline inclusions but have distinctive U-shaped configurations (Figure 5D, m).

While sperm of Rhipiphoridae have accessory bodies (Figure 5C, asterisk) that resemble those of sperm from a member of the meloid subfamily Nemognathinae (Figure 5B, asterisk), spermatozoa of meloid species in the subfamily Meloinae (Figure 5A, asterisk) more closely resemble sperm of *E. laboulbenei* (Figure 5D, asterisk) in lacking conspicuous accessory bodies.

In previous publications, a marked ultrastructural difference between Strepsiptera and other neuropteroid orders had been noted in the flagellar axoneme. The accessory tubules or peripheral singlets of the flagella in the Strepsipteran examined had 16 protofilaments (as do other neuropteroid orders) and had been described as having an incomplete “wall” resulting in a reniform or kidney shape that had not been observed in any other insect order [11,15]. This was considered the most compelling synapomorphy uniting the Strepsiptera. However, this structural feature of the singlets has been considered a function of the stage of sperm development and does not seem to persist in more mature accessory tubules [17]. The circular forms of singlet or accessory tubules of the *Eoxenos* axoneme of mature sperm do not match the reniform shapes of these tubules reported for the immature sperm of *Stylops* sp. [11]—a species that was incorrectly identified and is actually *Xenos* sp.

The lack of conspicuous accessory bodies has been a feature noted for the sperm of all strepsipteran species (families Xenidae, Elenchidae, Halictophagidae) examined in previous reports [11,13,14,16,18]. The flagella of the sperm of *E. laboulbenei* in the family Mengenillidae likewise do not have discrete accessory bodies (Figure 5D, asterisk). Interestingly, absence of accessory bodies is a sperm character shared with members of the entire order Diptera [15].

Despite an earlier claim [11] that the sperm axoneme of a Strepsipteran (*Xenos* sp.) lacked both accessory bodies and “intertubular material”, a more recent publication [1] claimed that *Xenos* axonemes have both intertubular material and accessory bodies. This claim about the presence of distinct accessory bodies, however, is debatable based on careful examination of figures in this publication [1].

As observed for representative members of other strepsipteran families, the mitochondrial derivatives of *E. laboulbenei* do not have the conspicuous, well-defined crystalline inclusions [13,14,16,18]. Also like all other strepsipterans whose sperm features have been described, the mitochondrial derivatives of *Eoxenos* have distinctive U-shaped configurations (Figure 5D, m).

## 4. Conclusions

### 4.1. Distinctive Physiological Demands of Strepsipteran Sperm May Be Reflected in Sperm Structure

Considering the highly simplified copulatory mechanism of Strepsiptera associated with the sessile, larviform adult females in the suborder Stylopidia, selective pressures on sperm size, structure, and function must presumably be singularly distinct from insects whose sexually mature females are motile and not larviform as in Mengenillidae (suborder Mengenillidia). Sperm is inserted in the brood canal opening of the extruded cephalothorax in all the endoparasitic females in the suborder Stylopidia [3,19], while in Mengenillidae (one of the two families in the primitive suborder Mengenillidia), traumatic insemination takes place where the male pierces any part of the free-living female to insert the sperm [20].

In Stylopidia the sperm moves from the brood canal opening to the brood canal, thence to the genital pores that open into the body of the female where the oocytes lie free in the body cavity. The first instar larvae move out of the body cavity of the viviparous female via the genital pores, brood canal and out to the open by the brood canal opening [21].

In Mengenillidae there is no special opening or passage for sperm; it moves directly into the body cavity to fertilize the oocytes that, as in Stylopidia, lie free in the body cavity. The genital pore in Mengenillidae is an opening only for the exit of the free-living first instar larva. Hence the distinctive, characteristic forms of the strepsipteran flagella [14] in addition to the absence of alignment and orientation among the mature cells in the testis may reflect the special physiological demands faced by sperm at the time of fertilization. 

### 4.2. Sperm Structure and Phylogeny

Ultrastructural features of insect sperm present a vast diversity of forms that presumably reflect the phylogenetic relationships among insects [2,15]. Morphological comparisons of sperm from four of the eight families of Strepsiptera have included comparisons between members of this distinctive order of parasitic insects and a limited number of species representing other holometabolous insect orders. The comparisons of sperm structure, in this manuscript as well as in earlier manuscripts, have offered limited support for a relationship between Coleoptera and Strepsiptera [1,11,18]; in some cases sperm ultrastructure has even been marshaled to offer tenuous support for the hypothesis that Diptera and Strepsiptera are sister groups [15]. Whiting *et al*. [22] published a cladistics study supporting this same sister-group relationship between the two orders, but this claim has now firmly been refuted; and genomic evidence firmly supports Strepsiptera being the closest living relatives of Coleoptera [6,7,23,24,25,26,27,28].

## References

[1] Dallai R., Beani L., Kathirithamby J., Lupetti P., Afzelius B.A. (2003). New findings on sperm ultrastructure of *Xenos vesparum* (Rossi) (Strepsiptera, Insecta). Tissue Cell.

[2] Jamieson B.G.M. (2011). The Ultrastructure and Phylogeny of Insect Spermatozoa.

[3] Kinzelbach R.K. (1971). Morphologische Befunde an Fächerflüglern und ihre phylogenetische Bedentung Insecta: Strepsiptera.

[4] Kathirithamby J. (2009). Host-parasitoid associations in Strepsiptera. Annu. Rev. Ent..

[5] McMahon D.P., Hayward A., Kathirithamby J. (2011). The first molecular phylogeny of Strepsiptera (Insecta) reveals an early burst of molecular evolution correlated with the transition to endoparasitism. PLoS One.

[6] McKenna D.D., Farrell B.D. (2010). 9-genes reinforce the phylogeny of holometabola and yield alternative views on the phylogenetic placement of Strepsiptera. PLoS One.

[7] Niehuls O., Hartig G., Grath S., Pohl H., Lehmann J., Tafer H., Donath A., Krauss V., Eisenhardt C., Hertel J. (2012). Genomic and morphological evidence converge to resolve the enigma of Strepsiptera. Curr. Biol..

[8] Böving A.G., Craighead F.C. (1931). An illustrated synopsis of the principal larval forms of the order Coleoptera. Entomol. Am..

[9] Kathirithamby J. (1989). Review of the order Strepsiptera. Syst. Entomol..

[10] Kinzelbach R.K., Pohl H., Dathe H.H. (2003). Ordnung Strepsiptera, Fächerflüger. Wirbellose Tiere. 5. Teil: Insecta.

[11] Afzelius B.A., Dallai R. (1994). Characteristics of the flagellar axoneme in Neuroptera, Coleoptera and Strepsiptera. J. Morph..

[12] Heming B.S. (2003). Insect Development and Evolution.

[13] Carcupino M., Mazzini M., Olmi M., Kathirithamby J. (1993). The spermatozoon of *Halictophagus chilensis* Hofmann (Strepsiptera, Halictophagidae). Boll. Zool..

[14] Kathirithamby J., Carcupino M., Mazzini M. (1993). Comparative spermatology of four species of Strepsiptera and comparison with a species of primitive Coleoptera (Rhipiphoridae). Int. J. Insect Morph. Embryol..

[15] Jamieson B.G.M., Dallai R., Afzelius B.A. (1999). Insects: Their Spermatozoa and Phylogeny.

[16] Mazzini M., Carcupino M., Kathirithamby J. (1991). Fine structure of the spermatozoon of the Strepsipteran *Xenos moutoni*. Tissue Cell.

[17] Carcupino M., Profili G., Kathirithamby J., Mazzini M., Jamieson B.G.M., Ausio J., Justine J.-L. (1995). Sperm ultrastructure of *Xenos vesparum* (Rossi) and its significance in the taxonomy and phylogeny of Strepsiptera (Insecta). Advances in spermatozoal phylogeny and taxonomy. Mémoires du Muséum National d’Histoire Naturelle.

[18] Kathirithamby J., Carcupino M., Mazzini M. (1992). Ultrastructure of the spermatozoon of *Elenchus japonicus* and its bearing on the phylogeny of Strepsiptera. Tissue Cell.

[19] Beani L., Giusti F., Mercati D., Lupetti P., Paccagnini E., Turillazzi S., Dallai R. (2005). Mating of *Xenos vesparum* (Rossi) (Strepsiptera, Insecta) revisited. J. Morph..

[20] Silvestri F. (1943). Studi sugli “Strepsiptera” (Insecta). III. Descrizione e biologia di 6 specie italiane di *Mengenilla*. Boll. Lab. Zool. Gen. Agric. Portici..

[21] Kathirithamby J. (2000). Morphology of the female Myrmecolacidae (Strepsiptera) including the *apron*, and an associated structure analogous to the peritrophic matrix. Zool. J. Linn. Soc..

[22] Whiting M.F., Carpenter J.C., Wheeler Q.D., Wheeler W.C. (1997). The Strepsiptera problem: Phylogeny of the holometabolous insect orders inferred from 18S and 28S ribosomal DNA sequences and morphology. Syst. Biol..

[23] Ishiwata K., Sasaki G., Ogawa J., Miyata T., Su Z.-H. (2011). Phylogenetic relationships among insect orders on three nuclear-coding gene sequences. Mol. Phylogenet. Evol..

[24] Friedrich F., Beutel R.G. (2010). Goodbye Halteria? The thoracic morphology of Endopterygota (Insecta) and its phylogenetic implications. Cladistics.

[25] Longhorn S.J., Pohl H., Vogler A.P. (2010). Ribosomal protein genes of holometabolan insects reject the Halteria, instead revealing a close affinity of Strepsiptera with Coleoptera. Mol. Phylogenet. Evol..

[26] Wiegmann B.M., Trautwein M.D., Kim J.-W., Cassel B.K., Bertone M.A., Winterton S.L., Yeates D.K. (2009). Single-copy nuclear genes resolve the phylogeny of the holometabolous insects. BMC Biol..

[27] Hayward D.C., Truman J.W.H., Bastiani M.J., Ball E.E. (2005). The structure of the USP/PXR of *Xenos pecki* indicates that Strepsiptera are not closely related to Diptera. Dev. Genes Evol..

[28] Rokas A., Kathirithamby J., Holland P.W.H. (1999). Intron insertion as a phylogenetic character: The *engrailed* homeobox of Strepsiptera does not indicate affinity with Diptera. Insect Mol. Biol..

